# Genetic Divergence of Thai Indigenous Pigs from Three Distinct Geographic Regions Revealed by Microsatellite Marker Analysis

**DOI:** 10.3390/ani13040625

**Published:** 2023-02-10

**Authors:** Kamon Chaweewan, Prapas Mahinchai, Sornchai Kongsook, Surasak Soponchit, Phuree Weerasamith, Wiranphat Awiruttapanich, Pakhawan Prapawat, Warocha Jamparat, Thitawat Chanthaworn, Natinee Rattanamahavichai, Sarisa Weangchanok, Siwaret Arikit, Monchai Duangjinda, Kunya Tuntivisoottikul, Chanporn Chaosap, Kanya Jirajaroenrat

**Affiliations:** 1 Interdisciplinary Graduate Program in Genetic Engineering and Bioinformatics, Kasetsart University, Bangkok 10900, Thailand; 2 Bureau of Animal Husbandry and Genetic Improvement, Department of Livestock Development, Pathum Thani 12000, Thailand; 3 Veterinary Biologics Assay and Research Center, Pakchong, Nakhon Ratchasima 30130, Thailand; 4 Bureau of Veterinary Biologics, Pakchong, Nakhon Ratchasima 30130, Thailand; 5 Department of Agronomy, Faculty of Agriculture at Kamphaeng Saen, Kamphaeng Saen Campus, Kasetsart University, Nakhon Pathom 73140, Thailand; 6 Department of Animal Science, Faculty of Agriculture, Khon Kaen University, Khon Kaen 40002, Thailand; 7 Department of Agricultural Education, School of Industrial Education and Technology, King Mongkut’s Institute of Technology Ladkrabang, Bangkok 10520, Thailand; 8 Department of Animal Technology and Fishery, School of Agricultural Technology, King Mongkut’s Institute of Technology Ladkrabang, Bangkok 10520, Thailand

**Keywords:** Thai indigenous pig, microsatellite markers, genetic polymorphism, population structure, phylogenetic tree

## Abstract

**Simple Summary:**

Thai indigenous pigs (TIPs) have long been associated with the socioeconomics of Thailand’s rural communities. They have been raised mainly in three different geographic regions of Thailand: north, northeast, and south. Most of the pigs have a black coat color. However, their genotypes have not been clarified. It is important to understand the genetic background to find an appropriate strategy for sustainable breeding and conservation. The TIPs from the three regions were genetically characterized in comparison. Overall, the TIPs showed a high diversity of microsatellite alleles. They differed significantly from European and Chinese breeds. However, the TIPs are at risk of genetic erosion and are losing genetic diversity due to the integration of local wide boars and the lack of a breeding plan. An appropriate breeding program for Thai pig populations is urgently needed to maintain these indigenous pig populations.

**Abstract:**

Thai indigenous pigs (TIPs) are important genetic resources. Crosses with exotic pig breeds and wild boars may cause genetic losses. To date, the physical characteristics of TIPs have been inconsistent. The classification of TIPs by genetic information is needed to pursue an appropriate conservation program. In this study, the genetic diversity, cluster analysis, and phylogenetic relationship of TIPs were investigated using twenty-nine pig microsatellite markers. Blood samples were collected from TIPs from three regions of Thailand: north (NT, *n* = 118), northeast (NE, *n* = 61), and south (ST, *n* = 75). The mean total number of distinct alleles and the effective number of alleles per locus were 11.851 and 5.497, respectively. The mean observed heterozygosity (Ho) and mean expected heterozygosity (He) were 0.562 and 0.837, respectively. The F values of the microsatellite loci were positive under Hardy–Weinberg Equilibrium at *p* < 0.001, with overall mean values of Fis, Fit, and Fst of 0.247, 0.281, and 0.046, respectively. A total of 5, 5, and 17 private alleles were found at frequencies greater than 0.050 in the NT, NE, and ST pigs, respectively. Three optimal clusters (*K* = 3) were proposed within the TIP populations. Pigs from the NT and NE regions were mixed in two clusters, while members of the ST region were clearly separated. The phylogenetic tree confirmed that the pigs from NT and NE were each divided into two subgroups, while the pigs from ST were clustered into one group. A microsatellite analysis revealed the high genetic diversity of the TIP populations and confirmed the genetic divergence of the TIPs from the European and Chinese breeds. A genetic admixture of the TIP with the local wild boars was detected.

## 1. Introduction

Pigs have been one of the most important protein sources for human consumption worldwide since ancient times. Analyses of animal bones from various archeological sites in Thailand have indicated that pigs were domesticated around the year 4000 BP [1]. It is believed that pig farming was associated with the rural lifestyle of local farmers. Pigs were raised in the backyard for personal consumption during ritual events and occasional ceremonies [2]. Thai indigenous pigs (TIPs) could be descended from both Chinese and local Southeast Asian ancestors, as inferred from ancient pig DNA [3]. TIPs typically have a black coat color, with occasional white markings on the nose, belly, and leg tips. They were previously classified into four groups based on their physical characteristics combined with their geographic regions of origin. The lower Northeastern Thai pigs “Raad”, sometimes called “Kadon”, have the smallest mature body weight (60–70 kg) while the Northern Thai pigs “Kwai” are the largest (130–150 kg). The indigenous pigs from the upper northeastern region called “Puang” and those from the western to southern regions called “Hainan” have an intermediate body weight of 120–130 kg and 110–120 kg for Puang and Hainan pigs, respectively [4]. TIPs can digest low-cost agricultural wastes and byproducts, some herbaceous native plants, and farmers’ leftover food [5,6]. TIPs have a low meat yield and thick back fat [4]. The meat is preferred by local consumers because it has special flavor and texture characteristics [7].

After intensive commercial pig production with the selection of high-performance pig breeds, European pigs such as Large White, Landrace, and Duroc were introduced into Thailand [4]; therefore, TIPs have decreased drastically. Currently, TIPs are observed as a small nuclei in rural villages mainly concentrated in the northern, northeastern, and southern regions of Thailand [8]. In addition, the Chinese Meishan breed was introduced to rural pig farmers by the Thai Department of Livestock Development (DLD) to improve the reproduction rate [9]. Recently, it has become difficult to identify breed-specific traits based on appearance and regions of origin due to the crossbreeding of exotic breeds and the translocation of native pigs. There is a risk that TIP genetic diversity will decrease and useful and desirable traits will be lost. In addition, the allocation of animals for unplanned crosses by farmers may lead to a loss of the genetic pool. Therefore, an attempt to understand the genetic diversity of today’s TIPs is of utmost importance to formulate recommendations for sustainable genetic management and conservation.

Microsatellites, also called Simple Tandem Repeats (STRs) or Simple Sequence Repeats (SSRs), are widely used to reveal genetic diversity and relationships in various livestock. They exhibit high polymorphism and are extremely useful markers for comparative studies of genetic variation, parentage assessment, traceability, gene flow, and hybridization studies [10,11,12]. They have been used to study biodiversity and the conservation of commercial, indigenous, and rare pig breeds worldwide [13]. In Asia, genetic diversity has been analyzed using microsatellite markers in Chinese native pigs [14,15,16,17,18], Korean black pigs [19], Taiwanese pigs [18], Indian native pigs [20], Filipino black pigs [21], and Vietnamese native pigs [22]. In Thailand, the study of Northern Thai pig populations using 14 porcine microsatellite markers revealed the distinct genetic relationship with European breeds [23]. Using 12 microsatellite markers, Northeastern and Southern Thai pigs showed closer genetic relatedness to Chinese breeds than to wild boars [24]. Using 26 microsatellites, another group of Northern Thai pigs showed a close relationship with local wild boars and Chinese breeds but diverged from European breeds [25]. In addition, Northern Thai hill tribe pigs with a complex appearance were classified into three clusters based on 15 microsatellite markers [26]. However, the differences in the selected microsatellite markers and pig populations used in previous reports make it difficult to plan an appropriate breeding strategy.

In this study, we aimed to investigate the genetic diversity, population cluster, and phylogenetic relationship of TIPs using the total 29 loci of the porcine microsatellite markers recommended by ISAG/FAO [27,28]. We collected samples from indigenous pigs from all three major geographic regions of Thailand (north, northeast, and south) and assumed that they represented the entire TIP population. The results will be used to develop a policy and system for sustainable genetic management, conservation, and utilization of indigenous pigs in Thailand.

## 2. Materials and Methods

### 2.1. Sample Collection and Populations

**Figure 1:**
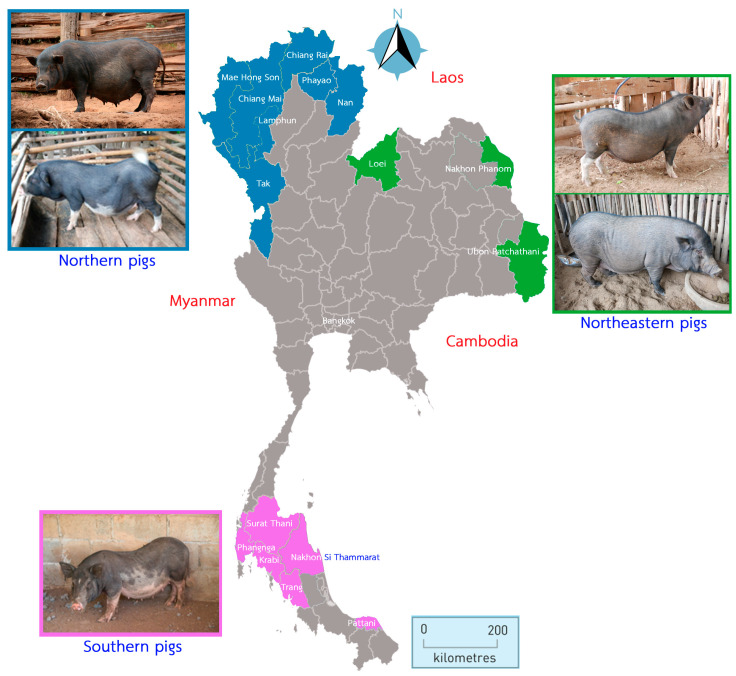
The Thai map shows the locations of the 16 provinces where indigenous pig samples were collected. The provinces of the three regions are color coded: north (blue), northeast (green), and south (purple). The physical characteristics of Thai indigenous pigs are shown next to the region of origin. The distance scale is in kilometres and the direction is shown.

Three TIP populations from the northern (NT, *n* = 118), northeastern (NE, *n* = 61), and southern (ST, *n* = 75) regions of Thailand were used for this study. Samples were collected in 16 provinces. All TIPs were raised in backyard husbandry and fed with agricultural byproducts. Blood samples were collected from healthy males and females at approximately 1 year of age. The samples were collected from 1–5 households per village and 1–2 pigs per household. They were not related. The sample animals were selected based on their origin and visible morphological characteristics (Figure 1). The NT pigs had two physical characteristics: (1) medium body size, black coat color with small ears and long, small nose and (2) medium body size, black coat color with small ears and white color on snout, leg, belly, and tail tip. The two morphological characteristics were also observed in the NE pigs: (1) medium size, black coat color with medium sized ears and white markings on nose, belly, leg, and tail tip and (2) large size, black coat color with small- to medium-sized ears and a long nose. The ST pigs had the largest body size compared to the NT and NE pigs, as well as a black body color with white markings on the nose, belly, and tail tip; a bent back; and a swag belly.

The NT pig samples were collected from hill tribal farmers in the Chiang Mai (CM, *n* = 5), Chiang Rai (CR, *n* = 15), Lamphun (LP, *n* = 11), Mae Hong Son (MH, *n* = 42), Nan (NA, *n* = 9), Phayao (PY, *n* = 27), and Tak (TK, *n* = 9) provinces at an altitude of >500 m above sea level near the border with Myanmar. The NE pig samples were collected in the Loei (LE, *n* = 29), Nakhon Phanom (NP, *n* = 6), and Ubon Ratchathani (UB, *n* = 26) provinces in the Mekong River Basin and the border with Laos. Samples from the ST pigs were collected from the Southern part of Thailand, including the provinces of Krabi (KB, *n* = 10), Nakhon Si Thammarat (NS, *n* = 37), Pattani (PT, *n* = 2), Phangnga (PG, *n* = 16), Surat Thani (SR, *n* = 8), and Trang (TR, *n* = 2), which are located on the Malay Peninsula.

The pig samples from Duroc (DR, *n* = 9), Landrace (LR, *n* = 10), Large White (LW, *n* = 10), and a cross of Duroc × Pietrain, called Pak Chong 5 (PC, *n* = 10), were used as the European breed references. Meishan (MS, *n* = 9) was used as the Chinese reference breed. All exotic breed stocks were provided by the Swine Research and Development Center (SRDC), Bureau of Animal Husbandry and Genetic Improvement (BAHGI), DLD, Nakhon Ratchasima Province, Thailand [29]. Wild boar samples (WB, *n* = 10) from backyard farms in the Loei, Sakon Nakhon, and Ubon Ratchathani provinces were used as the Thai wild boar reference.

### 2.2. Preparation of DNA Samples

Blood samples were stabilized in EDTA. Genomic DNA was extracted using the silica gel method described by Myakishev et al. [30] and was stored at −80 °C for further analysis.

### 2.3. Microsatellite Genotyping

A total of 30 porcine microsatellite markers recommended by the International Society for Animal Genetics (ISAG) including 18 microsatellite loci (IGF1, S0002, S0005, S0026, S0090, S0101, S0155, S0226, S0228, S0355, SW24, SW72, SW240, SW632, SW857, SW911, SW936, and SW951) [27] and another 12 microsatellite loci (S0068, S0097, S0143, S0178, S0218, SW122, SW445, SW830, SW1828, SW2008, SW2406, and SW2410) [28] were used in this study. Loci with an amplification failure higher than 5% of samples were excluded. All loci represented most of pig chromosomes (Table 1). The selected primers were labeled with WellRED Dye-Labeled Phosphoramidites (Beckman Coulter, USA). The polymerase chain reaction (PCR) was performed in a 10 µL mixture consisting of 1 µL of a 10× PCR buffer, 1 µL of dNTPs, 0.8 µL of MgCl_2_, 0.1 µL of *Taq* polymerase, 4.1 µL of dH_2_O, 0.5 µL each of forward and reverse primers, and 2 µL of the DNA template. The polymerase reaction was performed according to the following protocol: initial denaturation at 95 °C for 5 min followed by 35 cycles of 95 °C 30 sec denaturation, 55–60 °C 30 sec annealing (depending on primer conditions), and 72 °C 2 min extension. The PCR was completed with a 5 min extension at 72 °C. Multiplex fluorescence-tagged PCR products were separated by capillary electrophoresis and analyzed using the GenomeLab™ GeXP Genetic Analysis System (Beckman Coulter, Indianapolis, IN, USA).

### 2.4. Statistical Analysis

The mean number of alleles (Na), number of effective alleles (Ne), observed heterozygosity (He), expected heterozygosity, and private alleles (Pa) of the TIP populations were calculated using GenAlEx v.6.5 [31]. The polymorphic information content (PIC) of the microsatellite markers was calculated using the equations proposed by Botstein et al. [32]. The levels of genetic differentiation proposed as F-statistics, including Fis, Fst, and Fit indices [33], were calculated with 999 permutations using FSTAT v.2.9.4 [34]. Hardy–Weinberg Equilibrium (HWE) probabilities were estimated for all microsatellite loci with Fisher exact tests using a Markov Chain Monte Carlo (MCHC) simulation with 1000 dememorizations followed by 100 batches and 1000 iterations per batch using GenePop v.4.7.5 [35,36]. A Principal Coordinates Analysis (PCoA) was conducted using the covariance matrix with data standardization and plotted using GenAlEx v.6.5. A model-based Bayesian clustering of populations was analyzed using an admixture model for 10,000 burn-in times and 10,000 replications of MCHC with STRUCTURE v.2.3.4 [37]. The possible number of genetic clusters (K) was calculated between 2 and 20. The optimal number of clusters was determined based on the value of the second-order rate of change in the likelihood function to K (Δ*K*) [38]. The relationships between TIPs, exotic breeds, and wild boar populations were estimated using allele frequency data and calculated as DA distance [39]. The dendrogram was generated by using an unweighted pair group method with arithmetic mean (UPGMA) [40] with bootstrap tests of 1000 replicates using the POPTREEW program [41]. The pairwise distance between individuals was calculated based on the proportion of shared alleles to total alleles for each pair of individuals. The radiated phylogenetic tree was reconstructed using the Neighbor Joining method (NJ) in the program MEGA X v.10.2 [42].

## 3. Results

### 3.1. Microsatellite Polymorphism

A total of 29 porcine microsatellite markers were successfully amplified. The microsatellite marker with >5% amplification failure (S0355) was excluded. However, the markers represented most of the porcine chromosomes (Table 1). A total of 254 individual TIPs from three regions were examined. In the three pig populations, the number of different alleles (Na) ranged from 6.667 (S0090) to 19.333 (S0002), whereas the number of effective alleles (Ne) ranged from 2.916 (SW951) to 11.305 (S0002). The mean total number of different alleles per locus was 11.851, while the mean total effective number of alleles per locus was 5.497. Polymorphic information content values (PIC) varied from 0.752 (SW951) to 0.958 (S0002). The mean value of the PIC for 29 microsatellite loci was 0.874. Among the loci, the SW951 locus had the lowest He value (0.649), but S0002 had the highest He value (0.905). The values of observed heterozygosity (Ho) varied from 0.376 (S0026) to 0.796 (SW72). The overall mean values of He and Ho for the entire population were 0.787 and 0.595, respectively. Among the microsatellite loci, S0002 showed the highest genetic polymorphism for the entire TIP population.

Wright’s F-statistics describe the degree of inbreeding effects of an individual within subpopulations (Fis), within the whole population (Fit), and between subpopulations and the total population (Fst). The Fis value ranged from 0.067 (SW72) to 0.451 (S0026). The Fit ranged from 0.114 (SW72) to 0.519 (S0026). The Fst value varied between loci and ranged from 0.013 (S0090) to 0.123 (S0026). The overall mean of Fis, Fit, and Fst were 0.247, 0.281, and 0.046, respectively. Locus S0026 had the highest F-statistic value, indicating a strong influence of inbreeding in the population. All the analyzed loci differed from HWE with high significance (*p* < 0.001) according to Fisher’s exact test. 

### 3.2. Genetic Diversity among Thai Indigenous Pig Populations

The genetic diversity of the three TIPs was evaluated (Table 2). The NT, NE, and ST pigs had a few different alleles (Na) per locus of 12.655, 11.759, and 11.241, respectively. The effective alleles (Ne) were 5.399, 6.014, and 5.095 in the NT, NE, and ST pigs, respectively. The mean He value of the TIP was 0.787 whereas the mean Ho value was 0.596. The measurement of heterozygosity deficiency indicates the degree of inbreeding within a population, which is reflected by the inbreeding coefficient (Fis). The Fis values of the populations NT, NE, and ST were in the same direction: 0.260, 0.221, and 0.254, respectively. The deviations from HWE were significant among the TIPs in this study (*p* < 0.001).

Private alleles (Pa) indicate the allelic specificity of the population. Only alleles with a mean allele frequency (MAF) greater than 0.050 are shown (Table 3). Among the TIP populations, the ST pigs had the highest number of Pa with a total of 17 alleles. Among these alleles, allele 130 of SW240 and allele 167 of S0143 were found, both with MAF = 0.107. It is noteworthy that two alleles occurred at frequencies greater than 20%: Allele 98 at SW72 (MAF = 0.207) and Allele 121 at S0178 (MAF = 0.333). The NT and NE pigs had only 5 Pa each. Among these alleles, allele 97 of S0026 (NT), allele 236 of S0228 (NE), and allele 115 of S0178 (NE) had a MAF between 0.100–0.199.

### 3.3. Population Cluster Analysis

**Figure 2:**
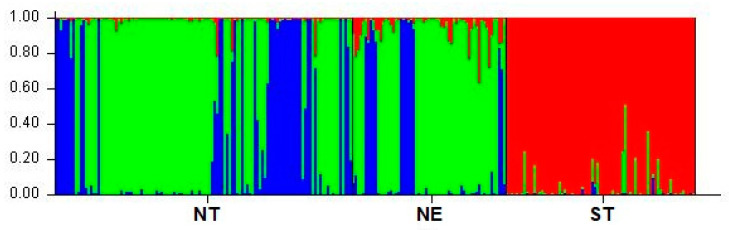
Individual membership of Thai indigenous pig populations at optimal cluster *K* = 3 estimated by the program STRUCTURE. Possible clusters are indicated by three colors: cluster 1 (red), cluster 2 (green), and cluster 3 (blue). The scale represents the proportion of membership in a total number of 100%. Three Thai indigenous pig populations originated from three regions (NT = north, NE = northeast, and ST = south).

The most probable number of ancestral populations of TIPs was evaluated from *K* = 2 to *K* = 20 using the admixture model analyzed with the STRUCTURE algorithm. After Δ*K* analysis, the optimal number of clusters was *K* = 3 (Figure 2). With *K* = 3, which was calculated as the most likely number of ancestral populations to explain the observed genetic variability in the three groups of Thai indigenous pigs studied, there should be three ancestral populations. The proportion of membership from the allocation test of individuals of the TIP is shown (Table 4). Most ST pigs belonged to cluster 1 (red), namely 95.7%. A large proportion of NT pigs (65.5%) and NE pigs (73.7%) were in cluster 2 (green). The rest of the NT and NE pigs were in cluster 3 (blue) with a percentage of 33.3% and 21%, respectively.

### 3.4. Genetic Relationship of Pig Populations

The pairwise genetic distances between TIP, the European breeds, the Chinese breed, and the wild boar populations were analyzed using Nei’s DA distance (Table 5). The lowest genetic distance was 0.162 between the NT and NE pigs. The genetic distances between the ST and NT (0.261) and ST and NE (0.250) pigs were approximately the same. The Chinese breed population (MS) was genetically more closely related to the NT pigs than to the NE and ST pigs, with distance values of 0.575, 0.625, and 0.769, respectively. The largest genetic distance was found to be 0.909 between the MS and LW pigs.

**Figure 3:**
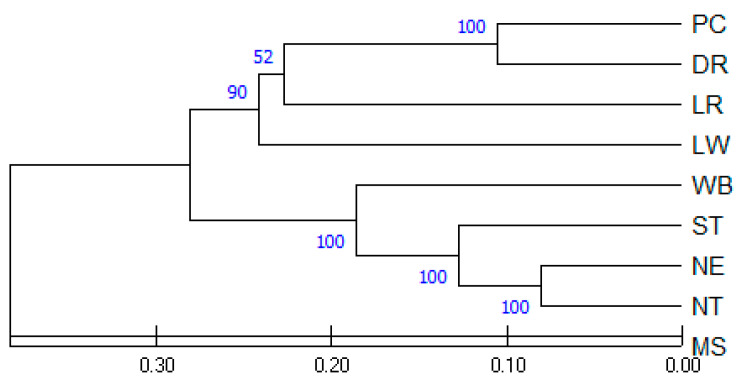
UPGMA dendrogram of nine pig populations reconstructed from Nei’s genetic distance. Branch lengths are proportional to genetic distances. Bootstrap tests with 1000 replicates are given as percentages at the nodes. Pig populations: PC = Pak Chong 5 crossbred; DR = Duroc; LR = Landrace; LW = Large White; WB = Thai wild boar; NE = Northeastern Thai pig; NT = Northern Thai pig; ST = Southern Thai pig; MS = Meishan.

The relationships among the nine pig populations were plotted in a dendrogram using the UPGMA algorithm (Figure 3). A microsatellite-based dendrogram grouped the nine pig populations into two main groups. The first group of MS formed a distinct branch that had less similarity to the other pig populations. The second group was further divided into two subgroups; the first subgroup included the four commercial breeds: LW, LR, DR, and PC, while the second subgroup included the ST, NE, and NT pigs and WB. The UPGMA dendrogram in the current study showed a close relationship between the TIPs and WB while they were different from the European commercial breeds and Chinese breed MS. The WB, which was included in the same subgroup of TIPs, showed a lower genetic distance from the TIPs (0.319–0.413) than the European breeds (0.589–0.652). The high bootstrap value (100%) between the TIPs strongly confirmed that the grouping of genetic distance by geographic criteria was correct. 

### 3.5. Principal Coordinate Analysis (PCoA)

**Figure 4:**
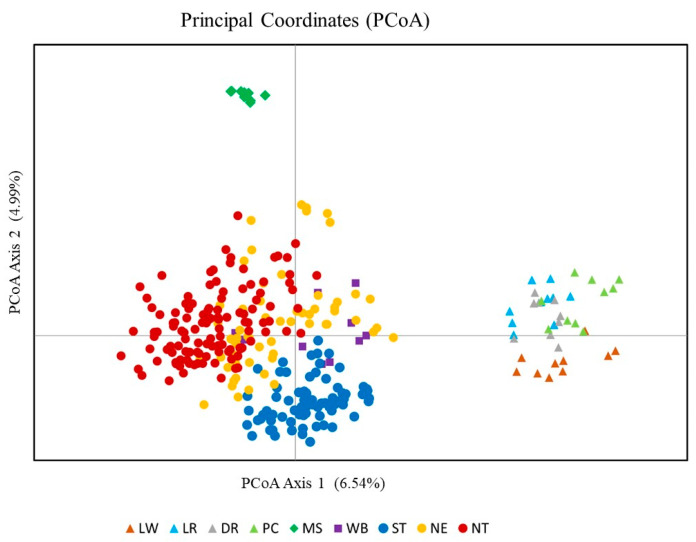
Principal coordinate analysis (PCoA) of individuals from nine pig populations. Pig populations: LW = Large White; LR = Landrace; DR = Duroc; PC = Pak Chong 5 crossbred; MS = Meishan; WB = Thai wild boar; ST = Southern Thai pig; NE = Northeastern Thai pig; NT = Northern Thai pig.

The plot of PcoA of the 312 individuals from nine pig populations was illustrated (Figure 4). Axes 1 and 2 of PcoA explained 11.53% of the total variance. Individuals of European breeds (LW, LR, DR, and PC) were aligned together. Individuals from MS were clearly separated from the Thai and European pig populations. The TIPs from the three regions (NE, NT, and ST) were positioned around the same coordinates. The Thai WB individuals overlapped with the TIP. 

### 3.6. Phylogenetic Tree of Individual Pigs

**Figure 5:**
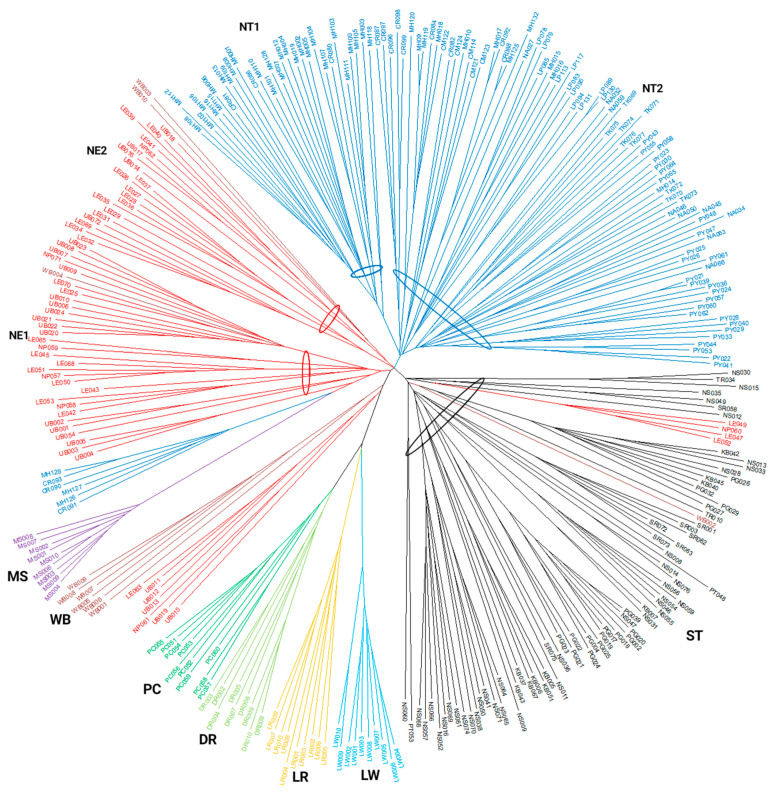
Neighbor Joining phylogenetic tree reconstructed from shared allele distances of 312 individual pigs. Pig populations are color coded: NT = Northern Thai pigs (blue); NE = Northeastern Thai pigs (red); ST = Southern Thai pigs (black); LW = Large White (light blue); LR = Landrace (yellow); DR = Duroc (yellowish green); PC = Pak Chong 5 crossbred (green); WB = Thai wild boar (brown); MS = Meishan (purple). NE1, NE2, NT1, and NT2 were subgroups. The oval line indicates the members of the group or subgroup. Sample identification numbers are shown. Provincial abbreviations: CM = Chiang Mai; CR = Chiang Rai; KB = Krabi; LE = Loei; LP = Lamphun; MH = Mae Hong Son; NA = Nan; NP = Nakhon Phanom; NS = Nakhon Si Thammarat; PG = Phangnga; PT = Pattani; PY = Phayao; SR = Surat Thani; TK = Tak; TR = Trang; UB = Ubon Ratchathani.

The Neighbor Joining phylogenetic tree was reconstructed to understand the relatedness of the 312 individual pigs based on shared allele distance matrices (Figure 5). The pigs were mainly located in their own populations. Four populations of European pig breeds, LW, LR, DR, and PC, were closely related, while the Chinese breed (MS) was genetically separated from the other pig populations. The Thai indigenous pigs were clearly divided into three main groups according to their geographic regions of origin: NT, NE, and ST. The NT pigs were divided into two subgroups: subgroup NT1 included most samples from Mae Hong Son (MH), and subgroup NT2 consisted of the most samples from the Chiang Rai (CR), Chiang Mai (CM), Lamphun (LP), Nan (NA), Tak (TK), and Phayao (PY) provinces. Some NT pigs from Mae Hong Son (MH129, MH126 and MH127) and Chiang Rai (CR090, CR091 and CR093) were separated from the main NT pig population and were located near the NE pig population. The NE pigs were clearly divided into two subgroups: subgroup NE1 contained most members from the Ubon Ratchathani (UB), Nakhon Phanom (NP), and some from Loei (LE) provinces, and subgroup NE2 contained most pigs from Loei province. However, some of the NE pigs (LE063, UB011, UB012, UB013, UB015, UB019, and NP061) shared alleles with Thai wild boars (WB). In addition, some of the WB pigs (WB003, WB004, and WB010) integrated into the NE pig population. Of the TIPs, only members of the ST pig population were within their own group. Some NE pigs (LE047, LE049, and LE052 from Loei province and NP060 from Nakhon Phanom province) shared alleles with the ST pigs.

## 4. Discussion

### 4.1. Characteristics of the Microsatellite Loci

In this study, all 29 microsatellite loci had high levels of allelic diversity. In general, microsatellite loci that have effective alleles ranging from 2 to 13 alleles per locus are preferred for population studies [43]. In our experiment, all loci had an effective allele value greater than two. The PIC is a parameter indicating the degree of genetic diversity, and a value greater than 0.5 is required to identify an individual [32]. All microsatellite loci showed high genetic variation, with a mean value of PIC of 0.874, which was higher than previous results studied by Charoensook et al. [25] (PIC = 0.789) and Gatphayak et al. [26] (PIC = 0.725) in Thai indigenous pigs. This could be due to the fact that our study was based on a larger sample size and examined a higher number of microsatellite loci. The use of a larger number of polymorphic loci led to a better result in the estimation of genetic distance [44]. In Hardy–Weinberg equilibrium (HWE), the expected heterozygosity (He) represents the theoretical genetic variability. In general, microsatellite loci with He values between 0.267 and 0.807 are suitable for use as genetic markers [43]. All microsatellite loci had He values greater than 0.6, indicating that they were sufficient for race identification according to Botstein et al. [32]. All Ho values of the studied loci were lower than the He values, indicating the effects of inbreeding within the entire indigenous pig population in Thailand. Overall, the 29 pig microsatellites used in the current study were desirable as genetic markers for evaluating the indigenous pig populations in Thailand.

Most allele sizes of the microsatellite loci of the TIPs were within the approximate range of those reported in European breeds [28], Northern Thai pigs [26], Indian pigs [20], and Korean black pigs [19] (Table S1). However, three loci of TIPs were different from those of Chinese black pigs: S0005 was larger and SW122 and SW911 were shorter than in Chinese black pigs [45]. Interestingly, three loci (S0226, S0097, and SW2406) had longer allele ranges compared to the exotic breeds, while two loci (SW2008 and S0143) had shorter allele ranges. The microsatellite loci with different allele ranges represent the specific loci of these populations. It is recommended that these loci be included in the microsatellite panel for the pig breeding program.

### 4.2. Genetic Relatedness among TIPs from the Three Geographic Regions

The mean number of effective alleles in the TIPs was 5.503, which was lower than the previous results of Yang et al. [24] (Ne = 6.30 in Southern Thai pigs and Ne = 7.09 in Northeastern Thai pigs). However, our results showed higher Ne values than those of Charoensook et al. [25] (Ne = 3.34–5.04 in Northern Thai pigs) and Gatphayak et al. [26] (Ne = 3.47 in Northern Thai pigs). The differences in polymorphic alleles between the studies may be influenced by the microsatellite panel, population size, and origin of the samples. The mean He values of the TIPs in this study were consistent with previous results in Thai pig populations, He = 0.71–0.79 [23,24,25,26]. The mean Ho values of the TIPs were approximately 20% lower than the mean He values, confirming that natural mating without a breeding program occurred in these populations. The TIPs clearly exhibited high heterozygosity. The Fis values of the TIPs ranged from 0.22–0.26, indicating a higher loss of heterozygosity compared to previous reports on Thai pig populations [25]. Some degree of inbreeding within the three TIP populations may have been due to the lack of breeding management by local farmers as well as the limited movement of animals in remote areas. The Fis values of TIPs were high and correlated with the occurrence of heterozygosity losses in individuals, which could be caused by panmixia within populations [18] or null alleles from population subdivisions [46]. Of the 27 private alleles identified in our study, our study clearly identified variable new alleles in the TIPs. However, none of them were identical to those reported by Charoensook et al. [25]. Our study provides only preliminary information on private alleles in TIPs and needs to be validated in pig populations before inclusion in a selection program. Therefore, the validation of these private alleles is required by the Thai Department of Livestock Development, which is responsible for the registration of livestock breeds in Thailand. It is possible to use the validated alleles as one of the identification tools to confirm the origin of the animals and to cluster the animals in the breed conservation plan.

Three different clusters were estimated for the TIPs according to their geographic regions of origin. However, only the Southern pigs are very homogeneous and associated with a single ancestral population. The phylogenetic tree confirmed the result of the population structure analysis and showed that the NE pigs were divided into two subgroups. The first group (NE1) was mainly from Ubon Ratchatani province, which is located in the lower northeastern region. The second group (NE2) consisted of most of the pigs from Loei province, which is located in the upper northeastern region. In addition, the indigenous pigs from Loei were raised in the areas near the border with Laos, separated by the small Hueang River, which is walkable in the dry season. Genetic transmission between indigenous pigs from Thailand and Laos across the border is possible. In the NT pig population, the population structure and phylogenetic tree suggested two subgroups. The first subgroup (NT1) from Mae Hong Son province was raised by the Musor hill tribe, which used this group of pigs for ritual ceremonies without mixing them with other pigs. In addition, the pigs were kept in a very remote area that was difficult to access. Therefore, crossbreeding was rare. The second subgroup (NT2) included the indigenous pigs from Chiang Mai, Chiang Rai, Lamphun, Nan, Tak, and Phayao. The area is easily accessible, allowing farmers to conveniently transport pigs across the province for breeding or rearing. Nevertheless, an admixture between the NT and NE pigs was observed. This phenomenon could have been due to the fact that the two regions are close to each other and therefore the pigs were transported across the region. Finally, the TIPs from the southern region were significantly different from those from the northern and northeastern regions. This could have been due to the geographic distance between the regions. In addition, the movement of animals to and from the Southern region of Thailand was restricted under the foot and mouth disease (FMD) free zone policy in accordance with the Department of Livestock Development regulations under the Animal Epidemics Act, B.E. 2558 [47]. According to this declaration, the ST pigs were certainly a segregated population.

### 4.3. Genetic Relationship of TIPs with Thai Wild Boars

In our study, the samples from WB were collected in the northeastern region, while those from Charoensook et al. [25] were collected in the northern region. The TIPs from our study were quite closely related to WB, confirming the observation of Yang et al. [24]. Among the TIP populations, the NE pigs were the closest relatives of WB. The close genetic relationship between the NE pigs and the local wild boars might have been due to the integration of wild boars into domestic pigs by farmers, which was enforced by consumer preferences in the region and commercial purposes of farmers [48]. To increase the commercial value of farm-raised pigs, the carcass characteristics demanded by the market must be improved. It has been shown that hybrids with good carcass characteristics, more leanness, and less fat can be produced by crossing wild boars with native sows [48]. In addition, farmers in rural areas generally kept their pigs in free-range systems, which led to natural mating with wild boars. As noted by Gama et al. [49], crossbreeding between the local wild boars and native pigs can occur, either by chance or through human management. Therefore, indigenous pigs are at risk of genetic erosion or loss, especially in the northeastern region.

### 4.4. Genetic Relationship of TIPs with Exotic Pigs

Compared with Asian indigenous pigs, the TIPs had higher Ne values than some native pigs, e.g., indigenous pigs from China (Ne = 2.53–3.76 [14] and Ne = 2.05–3.45 [50]), Taiwan (Ne = 2.39) [18], and Vietnam (Ne = 3.03–5.14) [22]. In addition, the Ne values of the TIPs were higher than those of European pigs as reported in Ukrainian pigs (Ne = 2.08–3.83) [51], several European breeds (Ne = 1.54–2.94) [52], Greek black pigs (Ne = 1.818–4.500) [53], and Portuguese pigs (Ne = 3.19–3.70) [54]. The populations of TIP had high effective alleles, suggesting high allelic polymorphism. This is likely due to the lack of a breeding program [25]. A measurement of heterozygosity deficiency provides information on the degree of inbreeding within a population, which is reflected in the inbreeding coefficient (Fis). The Fis values of the TIPs were high compared to the pig breeds of European origin bred under an appropriate breeding program. This result indicates a high rate of inbreeding in the populations of TIP and suggests that the Thai pigs were raised under an indiscriminate mating system. 

PCoA confirmed the genetic divergence of pig lineage in the same direction as the UPGMA dendrogram. The plots clearly distinguished pig samples of Thai origin including Thai wild boars from those of European breeds and the Chinese Meishan breed. Nei’s DA distance showed that Meishan pigs and European pigs were on different evolutionary paths. The genetic distance between the Thai pigs and the European commercial breeds was slightly smaller than that between the Thai pigs and the Chinese Meishan breed. The TIPs were clearly separated from the European breeds, which was consistent with the results of Charoensook et al. [25]. However, in our study, genetic divergence was found between the TIPs and the Chinese breed, in contrast to Charoensook et al. [25]. These inconsistent results could be explained by the different Chinese breeds used in the two studies. In the TIPs, we found that the pigs from Northeastern Thailand were more genetically related to the Chinese Meishan breed than the pigs from the northern and southern regions.

### 4.5. Implications and Prospects

Overall, the Thai indigenous pigs in our study showed higher levels of allelic diversity, heterozygosity, and richness in private alleles. There was evidence of a clear substructure in the Northeastern and Northern pig populations. The Northeastern pigs shared a significant proportion with local wild boars. A gradual admixture between Northeastern pigs and wild boars could result in the genetic introgression of wild boars into the gene pools of the Northeastern pig population, ultimately resulting in genetic loss. The wide diversity in genetic structure among the TIPs suggests that the three distinct geographic pig populations represent potential genetic resources for future genetic improvement and conservation. The registration of Thailand’s geographic pig breeds to promote breed conservation and market value should be considered. Further studies on the relationship between phenotypic traits and genotype are needed for a possible breed selection program. The evaluation of Thai pig populations by other approaches such as a maternal lineage study using mitochondrial DNA and a single nucleotide polymorphism (SNP) association with phenotypic traits are needed to better understand the genetic background of TIPs. The use of private alleles and TIP-specific microsatellite loci could be included in the relevant breeding program. In addition, a national policy for the conservation and sustainable use of TIPs should be urgently developed.

## 5. Conclusions

The porcine microsatellite markers used in this study showed a high degree of polymorphism and allelic diversity. Thai pig populations were relatively genetically specific. The indigenous pigs were classified into three main groups according to the three geographic regions of Thailand: north, northeast, and south. The NT and NE pig populations each contained two subgroups. The microsatellite panel was able to distinguish the Thai pigs from the exotic reference breeds. A genetic introgression with Thai wild boars was detected in the Northeastern pigs. The results of this study will be useful for the protection and conservation of the genetic resources of Thai indigenous pigs. In addition, the microsatellite panel used in this study can be used as one of the classification tools for Thai pigs. 

## Figures and Tables

**Table 1:** Genetic polymorphism of 29 microsatellite loci in Thai indigenous pig populations from three regions of Thailand.

Locus	SSC	Na	Ne	PIC	He	Ho	Fis	Fit	Fst	HWE
S0155	1q	10.333	4.552	0.857	0.776	0.659	0.151	0.169	0.021	***
SW1828	1	10.333	4.826	0.867	0.768	0.599	0.219	0.280	0.077	***
S0226	2q	12.000	4.129	0.847	0.757	0.570	0.247	0.273	0.035	***
SW240	2p	13.000	5.010	0.868	0.770	0.618	0.197	0.239	0.053	***
S0002	3q	19.333	11.305	0.958	0.905	0.740	0.182	0.208	0.033	***
SW72	3p	16.000	7.409	0.923	0.853	0.796	0.067	0.114	0.050	***
S0097	4	13.000	4.661	0.854	0.765	0.598	0.218	0.241	0.029	***
SW445	4	15.333	7.152	0.931	0.837	0.765	0.086	0.150	0.070	***
IGF1	5	10.333	4.200	0.874	0.759	0.529	0.303	0.363	0.086	***
S0005	5	15.333	7.945	0.920	0.872	0.701	0.196	0.214	0.022	***
S0228	6	11.667	6.639	0.919	0.845	0.526	0.378	0.408	0.049	***
SW122	6	10.667	3.730	0.809	0.706	0.527	0.254	0.281	0.036	***
SW2406	6	11.333	5.291	0.895	0.789	0.611	0.226	0.278	0.068	***
S0101	7	8.667	3.099	0.775	0.667	0.465	0.303	0.327	0.035	***
SW632	7	9.667	3.319	0.798	0.698	0.528	0.244	0.259	0.019	***
S0178	8	14.000	6.717	0.917	0.845	0.614	0.273	0.307	0.046	***
SW2410	8	13.667	8.208	0.935	0.878	0.666	0.242	0.266	0.032	***
SW911	9	9.667	6.132	0.913	0.834	0.723	0.133	0.174	0.048	***
SW830	10	12.333	5.541	0.895	0.809	0.539	0.334	0.365	0.047	***
SW951	10	8.000	2.916	0.752	0.649	0.465	0.284	0.301	0.023	***
SW2008	11	7.667	3.888	0.826	0.731	0.469	0.358	0.397	0.061	***
S0090	12	6.667	4.477	0.847	0.776	0.466	0.399	0.407	0.013	***
S0143	12	10.667	4.605	0.836	0.752	0.629	0.163	0.205	0.051	***
S0068	13	15.667	7.455	0.932	0.862	0.724	0.160	0.198	0.046	***
SW857	14	9.667	5.597	0.888	0.817	0.639	0.217	0.235	0.023	***
SW936	15	13.333	7.563	0.924	0.867	0.614	0.292	0.312	0.028	***
S0026	16	9.667	3.768	0.873	0.685	0.376	0.451	0.519	0.123	***
SW24	17	13.667	5.117	0.890	0.799	0.680	0.149	0.192	0.051	***
S0218	X	12.000	4.168	0.835	0.743	0.416	0.440	0.467	0.049	***
Mean		11.851	5.497	0.874	0.787	0.595	0.247	0.281	0.046	***

SSC = Sus scrofa chromosome; Na = number of different alleles; Ne = number of effective alleles; PIC = polymorphic information content; He = expected heterozygosity; Ho = observed heterozygosity; Fis = inbreeding coefficient related to subpopulations; Fit = inbreeding coefficient related to total population; Fst = genetic differentiation index; HWE = Hardy–Weinberg Equilibrium. *** *p <* 0.001.

**Table 2:** Genetic diversity among indigenous pig populations from three distinct regions of Thailand.

Population ^1^	Na	Ne	He	Ho	Fis	HWE
NT	12.655	5.399	0.778	0.574	0.260	***
NE	11.759	6.014	0.801	0.628	0.221	***
ST	11.241	5.095	0.781	0.586	0.254	***
Mean	11.885	5.503	0.787	0.596	0.245	***

^1^ NT = Northern Thai pigs; NE = Northeastern Thai pigs; ST = Southern Thai pigs. Na = average number of different alleles; Ne = average number of effective alleles; He = expected heterozygosity; Ho = observed heterozygosity; Fis = inbreeding coefficient; HWE = Hardy–Weinberg equilibrium. *** *p* < 0.001.

**Table 3:** List of the private alleles (in bp) in Thai indigenous pig populations ^1^ from three regions of Thailand.

Locus	NT	NE	ST
S0155	-	-	153 (0.060)
SW1828	-	-	-
S0226	-	-	209 (0.053)
SW240	-	-	130 (0.107)
S0002	-	-	-
SW72	-	-	98 (0.207)
SW445	-	-	-
S0097	223 (0.052)	210 (0.057)	-
IGF1	-	-	-
S0005	-	203 (0.092), 222 (0.057)	229 (0.053), 239 (0.080)
S0228	-	236 (0.189)	248 (0.073)
SW2406	-	-	242 (0.080)
SW122	-	-	-
SW632	-	-	-
S0101	224 (0.085)	-	-
S0178	-	115 (0.139)	121 (0.333)
SW2410	-	-	113 (0.053)
SW911	-	-	-
SW951	-	-	-
SW830	200 (0.097)	-	185 (0.053), 189 (0.067)
SW2008	-	-	110 (0.060)
S0090	-	-	-
S0143	-	-	167 (0.107)
S0068	-	-	-
SW857	-	-	133 (0.053)
SW936	-	-	123 (0.080)
S0026	97 (0.161), 107 (0.081)	-	-
SW24	-	-	-
S0218	-	-	178 (0.060)
Total	5	5	17

^1^ NT = Northern Thai pigs; NE = Northeastern Thai pigs; ST = Southern Thai pigs. Mean allele frequencies are in brackets.

**Table 4:** Proportion of membership of each Thai indigenous pig population in 3 clusters (*K* = 3).

Population ^1^	Cluster		
	1	2	3
NT	0.012	0.655	0.333
NE	0.053	0.737	0.210
ST	0.957	0.037	0.007

^1^ NT = Northern Thai pigs; NE = Northeastern Thai pigs; ST = Southern Thai pigs.

**Table 5:** Pairwise matrices of Nei’s DA distance among nine pig populations.

Population ^1^	NT	NE	ST	LW	LR	DR	PC	MS	WB
NT	-								
NE	0.162	-							
ST	0.261	0.250	-						
LW	0.587	0.593	0.516	-					
LR	0.595	0.568	0.566	0.482	-				
DR	0.540	0.477	0.522	0.504	0.487	-			
PC	0.548	0.474	0.506	0.461	0.421	0.211	-		
MS	0.575	0.625	0.769	0.909	0.835	0.860	0.825	-	
WB	0.383	0.319	0.413	0.652	0.639	0.589	0.611	0.726	-

^1^ NT = Northern Thai pigs; NE = Northeastern Thai pigs; ST = Southern Thai pigs; LW = Large White; LR = Landrace; DR = Duroc; PC = Pak Chong 5 crossbreed; MS = Meishan; WB = Thai wild boar.

## Data Availability

The data used to support the findings in this study are available from the corresponding authors upon request.

## Supplementary Materials

The following supporting information can be downloaded at: https://www.mdpi.com/article/10.3390/ani13040625/s1, Table S1: Allele range (base pairs) of microsatellites loci in pig populations from different origins.
