# Supplementary material for: Genetic Divergence of Thai Indigenous Pigs from Three Distinct Geographic Regions Revealed by Microsatellite Marker Analysis
Source: Animals (Basel). 2023 Feb 10;13(4):625. doi: 10.3390/ani13040625 (PMC9951687; doi:10.3390/ani13040625)
Supplement: Supplementary file 1 [file animals-13-00625-s001.zip › animals-2143250-supplementary.pdf]

Table S1. Allele range (base pairs) of microsatellites loci in pig populations from different origins.

Locus	Country/Origin of data					
	Thailand	Thailand	China	India	Korea	ISAG
S0155	137-169	132-166	116-158		145-168	150-166
SW1828	83-114					100-104
S0226	201-237*		166-198	185-205		181-205
SW240	86-130	88-114				96-115
S0002	182-246					190-216
SW72	98-122		90-118	100-112	95-134	100-116
S0097	208-249*		135-155			135-155
SW445	172-210					181-203
IGF1	190-210					197-209
S0005	203-257	201-246	134-168*	221-257	200-257	205-248
S0228	210-248					222-249
SW122	98-132		220-247*	110-124	116-138	110-122
SW2406	224-257*		117-131			117-131
S0101	197-226	203-213				197-216
SW632	150-174		148-173		151-190	159-180
S0178	102-130					110-124
SW2410	100-142					81-119
SW911	149-175		217-255*	157-175		153-177
SW830	169-216		149-173			149-173
SW951	120-141	120-132	123-133		123-145	125-133
SW2008	89-110*					148-170
S0090	239-254	226-249	227-253	243-251	200-260	244-251
S0143	151-175*					261-289
S0068	211-259			218-242		211-260
SW857	133-165	134-162	144-168		142-168	144-160
SW936	91-127		91-120		80-120	80-117
S0026	91-111				90-113	92-106
SW24	91-128	93-118		92-112	91-129	96-121
S0218	157-199			164-184		164-184
Reference	This Study	[22]	[42]	[16]	[17]	[25,26]

*Allele range differs from other reports.
